# Post-discharge stroke patients’ information needs as input to proposing patient-centred eHealth services

**DOI:** 10.1186/s12911-016-0307-2

**Published:** 2016-06-07

**Authors:** Nadia Davoody, Sabine Koch, Ingvar Krakau, Maria Hägglund

**Affiliations:** Department of Learning, Informatics, Management and Ethics, Health Informatics Centre, Karolinska Institutet, Tomtebodavägen 18 A, 171 77 Stockholm, Sweden; Department of Medicine, Karolinska Institutet, Solnavägen 1, 171 77 Stockholm, Sweden

**Keywords:** Information and communication technology, Health information system, eHealth services, Stroke, Rehabilitation, Patient experience, Patient journey

## Abstract

**Background:**

Despite the potential of eHealth services to revolutionize the way healthcare and prevention is provided many applications developed for patients fail to deliver their promise. Therefore, the aim of this study is to use patient journey mapping to explore post-discharge stroke patients’ information needs to propose eHealth services that meet their needs throughout their care and rehabilitation processes.

**Methods:**

Three focus groups with younger (<65 years) and older (> = 65 years) stroke patients were performed. Content analysis was used to analyse the data. Stroke patients’ information needs was explored using patient journey model.

**Results:**

Four main events (discharge from hospital, discharge from rehab clinic, coming home, and clinical encounters) and two phases (at rehab clinic, at home) have been identified in patients’ post-discharge journey. The main categories identified in this study indicate that patients not only need to have access to health related information about their care and rehabilitation processes but also practical guidance through healthcare and community services. Patients also have different information needs at different events and during different phases. Potential supportive eHealth services were suggested by the researchers considering different parts of the patients’ journeys.

**Conclusions:**

Patient journey models and qualitative analysis of patients’ information needs are powerful tools that can be used to improve healthcare from a patient perspective. As patients’ understanding of their illness changes over time, their need of more flexible support throughout the care and rehabilitation processes increases. To design appropriate eHealth services that meet patients’ information needs, it is imperative to understand the current care and rehabilitation processes and identify patients’ information needs throughout their journey.

## Background

Healthcare is shifting from a paternalistic model to a patient-centred care model. In the latter model the patients’ individual needs are in focus and healthcare is provided based on a partnership between patients and healthcare professionals [1]. Patient-centred care requires, therefore, an active involvement of patients throughout the care and rehabilitation processes as well as new ways of designing and developing health information systems and eHealth services. Studies have shown that the use of information and communication technology (ICT) has been increased in healthcare as it has a great potential impact on healthcare quality and patient involvement [2–5]. The use of ICT in healthcare has led to creation of new terms such as eHealth. The definition of eHealth varies depending on the context where the term is used [6, 7]. According to Eysenbach eHealth refers “to health services and information delivered or enhanced through the Internet and related technologies. In a broader sense, the term characterizes not only a technical development, but also a state-of-mind, a way of thinking, an attitude, and a commitment for networked, global thinking, to improve healthcare locally, regionally, and worldwide by using information and communication technology” [8]. Koch et al. provide a new description of eHealth in 2010: “We see eHealth not in the broader scope of encompassing the entire field of health and medical informatics but as a continuation of the fields of telemedicine and telehealth that in combination with the rising field of consumer informatics has the potential to revolutionize the way healthcare and prevention is provided, shifting the balance of power and responsibility from healthcare professionals to patients and citizens” [9]. Another term used in this study is e-services. According to Rowley “e-services are deeds, efforts or performances whose delivery is mediated by information technology (including the web, information kiosks and mobile devices)” [10]. In this study the term “eHealth services” refers to e-services within the health domain which can be used by patients and/or care providers. Despite the potential of eHealth services to revolutionize the way healthcare and prevention is provided, shifting the balance of power and responsibility from healthcare professionals to patients and citizens [9, 11], many applications developed for patients are either designed from a healthcare provider’s perspective or standalone health applications, e.g. mobile apps for activity tracking. A more balanced way for initiating eHealth service is to take patients’ experiences of the patient journey into account in the design process [12, 13]. To design eHealth services that provide patients with a holistic overview of their often fragmented care requires a deep understanding of their experiences of the patient journey. In service design [14], customer journey mapping is a tool used for gaining insight into customers’ experience of using a service. Costumer journey maps “illustrate the steps your customer(s) go through in engaging with your company, whether it be a product, an online experience, retail experience, or a service or any combination” [15]. This method has lately also been applied in healthcare to describe patients’ experiences [16, 17]. Patient journey refers to “the experiences and processes the patient goes through during the course of a disease and its treatment” [17]. The patient journey model gives a common picture of the processes and the way the patients experience their care and rehabilitation processes. In this study, post-discharge stroke patients’ information needs and their need for appropriate eHealth services throughout the patient journey is in focus.

### Stroke

Stroke patients suffer from physical, cognitive, and psychosocial disabilities and require care from many different healthcare professionals over a long period of time. Corbin and Strauss’ Chronic Illness Trajectory framework [18] describes the experience of chronic illnesses and provides a long-term perspective on a patient’s recovery process [19]. According to the trajectory framework, the experience, impact and needs change over time as the patient experiences of illness changes [20]. Access to information throughout the care and rehabilitation processes is a well acknowledged necessity and Foster et al. in a systematic review show that several attempts have been made to provide appropriate information to patients and their caregivers [21]. Despite this, stroke patients still experience unmet information needs [22]. In a previous study we explored the current care and rehabilitation processes in post-discharge stroke care in Stockholm, Sweden, from the care professionals’ perspective [23], and confirmed that there is still limited support for providing information to patients and their next-of-kin.

Understanding the patient journey is imperative to be able to develop eHealth services that provide necessary information and meet patients’ needs throughout their illness journey.

This study was performed within the Swedish research project “My Care Pathways” [24]. The aim of the project is to enable patients to monitor, own and manage their care process through innovative mobile eHealth services. The care process and information needs of three patient groups are explored within the project, namely; stroke patients, hip surgery patients [12], and lung cancer patients [13].

### Objective

The aim of this study is to explore post-discharge stroke patients’ information needs using patient journey mapping and to use patients’ perceived needs to propose eHealth services that can support them throughout their care and rehabilitation processes.

## Methods

Analysis of the patients’ information needs throughout their care and rehabilitation processes was done as the first stage of a user-centred design approach [25], actively involving stroke patients in the process. Qualitative research methods were used as a mean to get a deep understanding of patients’ experiences and information needs throughout the patient journey. Data was gathered through three focus groups [26]. The trajectory framework was used as guidance throughout the study and was a basis for the analysis of the stroke patients’ journey throughout the care and rehabilitation processes. The study obtained ethical approval from the Regional Ethics Committee in Stockholm on January 19th 2012 (2011/2093-31/5).

### Focus groups

Three focus groups with stroke patients were performed. Each focus group lasted approximately 2-3 h. The participants had mild physical, cognitive and/or psychosocial disabilities, were living in their own homes, were capable of handling computers and were able to give informed consent to participate in the study. The participants were recruited through patient organisations using purposive sampling [26]. Both older (> = 65 years) and younger (<65 years) stroke patients participated in the study. Both patients who suffered a stroke more than 10 years ago and patients with more recent strokes were included as participants. We consciously included patients with a longer illness-trajectory to be able to get their long-term perspective on the care and rehabilitation process. Despite the fact that stroke care health services may have changed considerably over the past 10 years, there is still a lack of eHealth services providing information and support to post-discharge stroke patients, and therefore patient experiences and needs are likely relevant even if they are not recent. An overview of participants is presented in Table 1.

**Table 1:** An overview of participants

Focus groups	The number of participants	Time from first stroke	The age range	Gender
Focus group 1	4	>10 years	65-85	Female: 2
				Male: 2
Focus group 2	4	>10 years	65-85	Female: 2
				Male: 2
Focus group 3	4	<10 years	30-64	Female: 3
				Male: 1

**Fig. 1:**
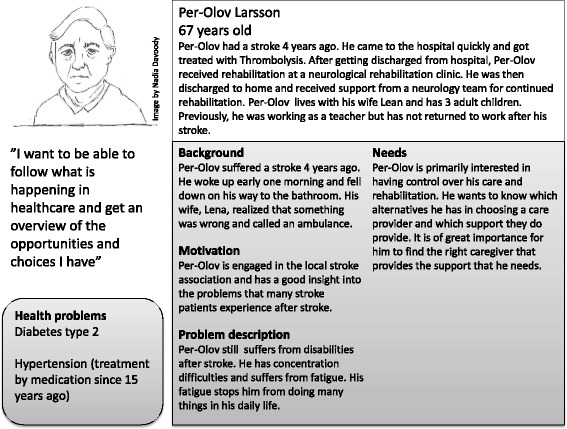
An example of a persona that gives a concrete and specific representation of a user group. The information is not that of a real person

A user-centred design [25] approach was applied, requiring an in-depth understanding of the users, their activities and needs as the starting point for any design activities. This study aims to provide this understanding using patient journey modelling as one tool. In addition, we created so called personas to represent our stroke patients. A persona is a concrete and specific representation of a user group and is used to create empathy and understanding for the users within the entire design team [27, 28]. In this study, the personas were used to facilitate discussions in the focus groups. A total of four personas were developed that covered different age groups, risk factors, disabilities and health problems as well as interest in using e-services and new technology to achieve a rich picture of how different stroke patients’ needs for design of eHealth services may differ. Figure 1 illustrates an example of a persona used in this study.

In scenarios that were discussed in the focus groups the persona characters were used to illustrate different situations and different problems they might experience. For example the scenario “home visit of the neurology team” was created to describe the problem “lack of overview of the rehabilitation process” experienced by the patient represented as persona in Fig. 1.

**Fig. 2:**
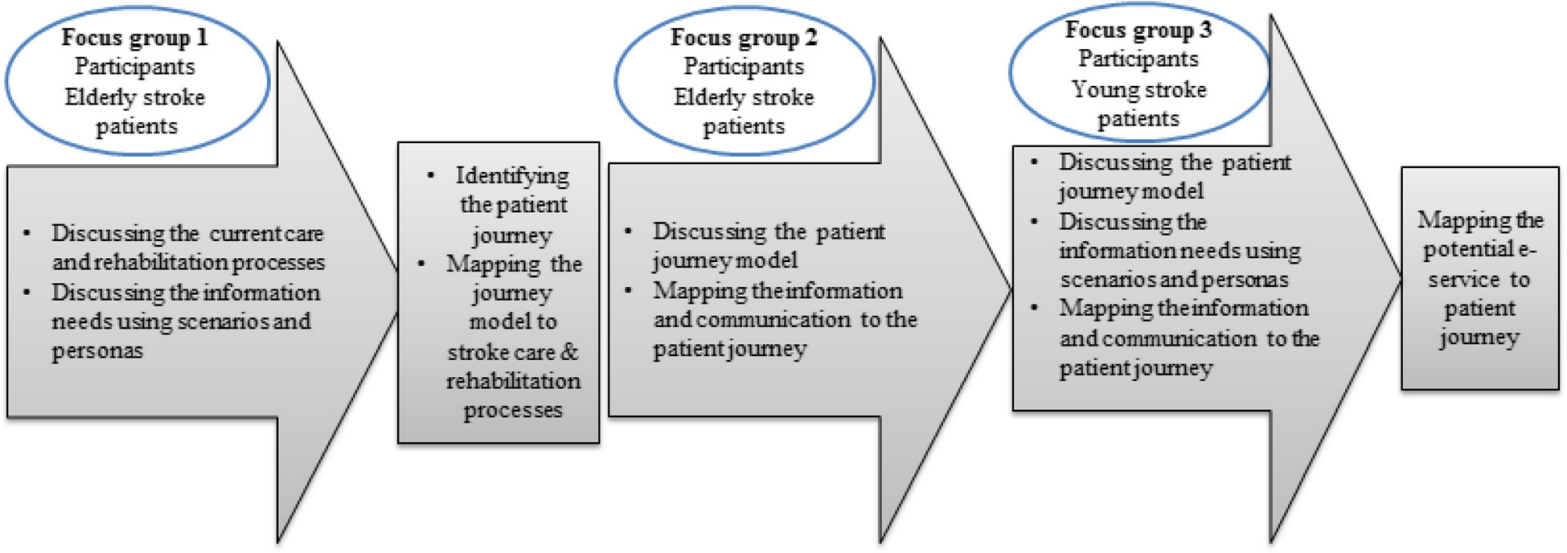
An overview of the process of analysing patients’ experiences and needs

Figure 2 illustrates an overview of the approach, including the topics discussed during each focus group and actions performed by the researchers in between and after the focus groups.

All focus groups were facilitated by a moderator and notes were taken by two researchers. The focus groups were audio recorded and transcribed verbatim. Content analysis [29] was used to identify the categories and themes. Two researchers independently reviewed the material from each focus group using an inductive approach [30]. Meaning units were identified, condensed and categories and themes were derived from the data and discussed with other members in the research group [26].

To identify the patients’ needs of eHealth services intended to provide them with necessary information, it is imperative to initially explore their information needs in post-discharge stroke care. Therefore we in this study identified different phases of the care and rehabilitation processes and presented these using a patient journey model. We then identified the patients’ information needs at different stages of the processes. Finally, we suggested the potential eHealth services including necessary functionalities that can support patients’ needs of information.

At the first focus group, different phases of the care and rehabilitation processes were discussed with the stroke patients. A patient journey model was then developed based on the flowchart of the stroke care chain in Stockholm County [23] as well as on the discussions during the first focus group. The model was presented to the group at the second and the third focus groups and the patients’ information needs were discussed considering the different phases of the journey. Finally, potential eHealth services proposed by the researchers were discussed with the participants. One of the potential eHealth services identified consisted of a rehabilitation plan. The specific information needs regarding the rehabilitation plan were elicited through further focus groups with other post-discharge stroke patients and results were presented by Lyckstedt [31] and Woldermariam [32].

## Results

Patients’ experiences and information needs vary at different phases of the process from the incidence of stroke to the subsequent care at home. In this study, we focus on the post-discharge stroke patients’ experiences and the results will be presented in form of a patient journey model, followed by an analysis of the patients’ information needs and supportive eHealth services proposed by the authors of this study.

### The post-discharge patient journey model

**Fig. 3:**
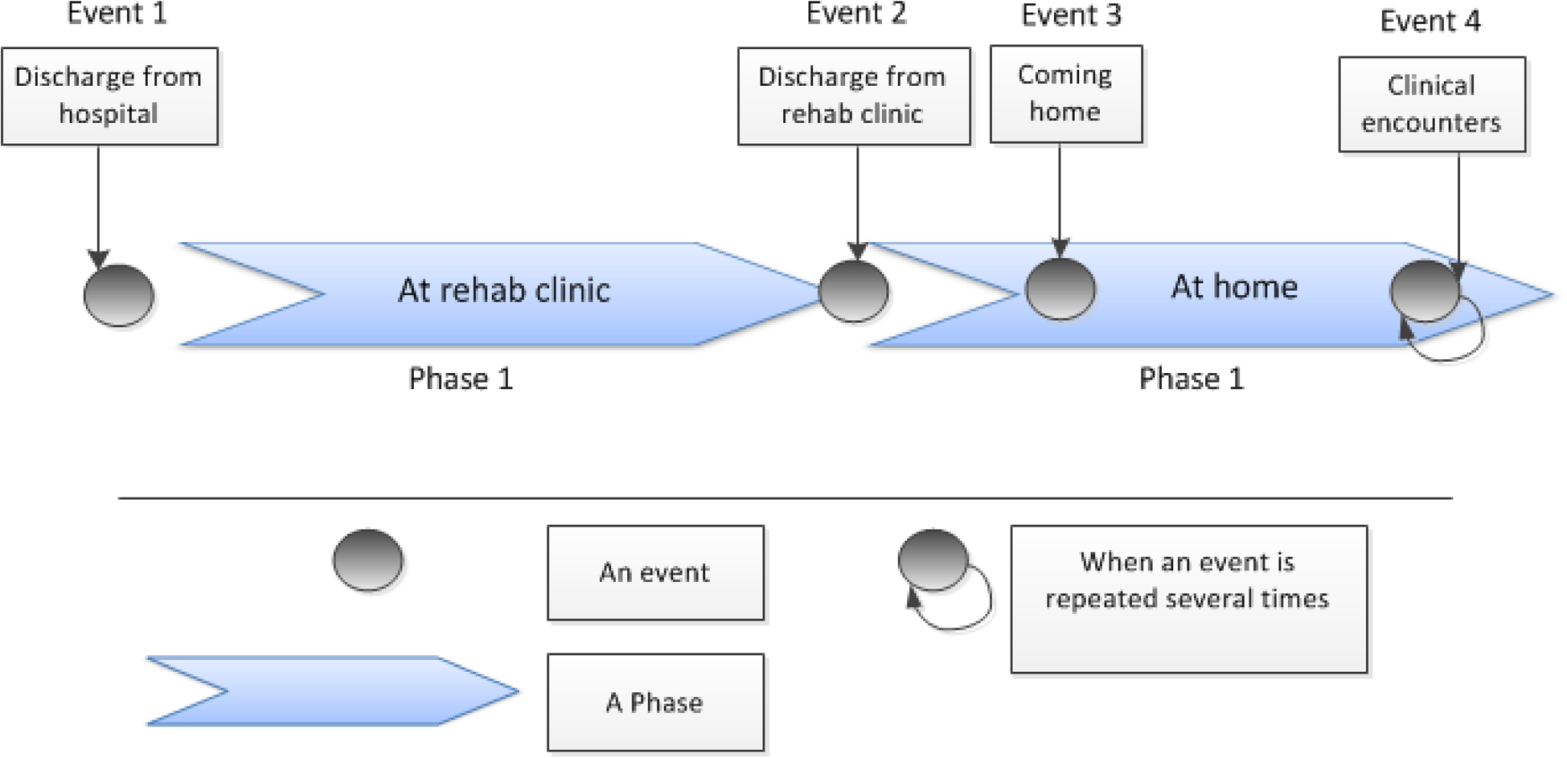
An overview of the post-discharge stroke patients’ journey

We distinguish between phases and events in the patient journey model (Fig. 3). A phase is extended over time and may incorporate several events. An event is a specific interaction between patient and healthcare, where information is created, shared or communicated. Patient journey models sometimes only include phases, but to use the patient journey as a basis for design of eHealth services, it is important to also map events, typically called touch points in customer journey maps [14].

The analysis of the patient journey model indicates that post-discharge stroke care can be divided into four main events and two phases (Fig. 3). Specific information such as discharge notes are created and shared with the post-discharge stroke patients at the following main events: *discharge from hospital*, *discharge from rehab clinic*. In addition a considerable amount of health related information communicated to the patients and their next-of-kin during the main events called *coming home* and *clinical encounters*. Two main phases in the post-discharge stroke patient journey identified in this study include *at rehab clinic* and *at home* in which patients receive care and rehabilitation over time.

### Analysis of patients’ experiences and needs throughout the post-discharge journey

The content analysis of data from the focus groups identified 5 essential categories with subcategories (Table 2).

**Table 2:** An overview of identified categories and subcategories

Categories	Subcategories
A holistic view of the care process	An overview of past events
	An overview of planned events
Understanding the illness	An overview of clinical information
	An overview of risk factors and disabilities
	Measurement and documentation of health related parameters
Collaboration with care providers	An overview of care providers’ contact information, their specialities and responsibilities
	Support for sharing of personal observations
Tracking the rehabilitation process	An overview of goals and planned activities
Practical guidance through healthcare and community services	Rights and responsibilities regarding e.g. continued rehabilitation, assistive devices and general information about health insurances
	An overview of patient associations and social networks

#### A holistic view of the care process

Currently, access to information is fragmented and patients receive information from many healthcare professionals in a variety of formats (oral, paper, and electronic) [23]. Patients experienced a lack of overview of necessary information in the fragmented health system.

*“I am the only one who has a complete picture in my fuzzy mind.”*

*“You want to see and follow what has happened- what I have done and who I have met.”*

##### An overview of past events

Participants in the study wished to have access to information from the discharge meeting at the hospital or the rehab clinic. They wished to be able to go through the information about e.g. their health condition at hospital, the diagnosis, treatments, lab results, and the care and rehabilitation they received. The participants expressed that they were only beginning to gradually capture and understand information when they returned home.

*“Yes, because then you can go back a bit when you want and feel that you have more energy.”*

In addition, some of the participants were interested in recording the discharge meeting as a memory support. Processing information during stressful event can be a challenge, and therefore it is important to be able to review the information later and written notes is not ideal for all patients – alternatives are needed to suit individual stroke patient’s needs.

##### An overview of planned events

Participants were interested to have access to the information from discharge meetings as it concerns what is planned for the patient at home.

*“It would be good to have it [the discharge note] at home and to read it in peace and quiet.”*

They were also interested in having access to information about their referrals and the units that receive the referrals. The participants were looking for an opportunity to monitor the information flow by knowing who has been informed and what will happen next.

*“It should be possible to search for information such as: where I have ended up and why I have not been contacted etc. For me, it took 6 months before I was contacted by my primary care centre.”*

*“You must know who you should contact, and if they have received information [about my stroke], and where the information is and why my caregivers do not receive the information…”*

Participants believed that the information about the time schedule for the neurology team’s home visits and appointments with other care professionals is of great importance as they will be able to track appointments throughout the care and rehabilitation processes.

*“At a later stage of the recovery, when you are more or less receiving care and rehabilitation from primary care, diary and calendar are more important.”*

*“But for relatives, I believe that calendar and information such as what times I have booked and when the neuro team do their home visits, are important.”*

#### Understanding the illness

Participants’ interest in having access to information about e.g. their medications, diagnosis, and disabilities indicates that gaining an understanding of their illness is of great importance for them.

##### An overview of clinical information

The clinical information is documented in the patient’s health record. Participants expressed that they need to have access to understandable information about their diagnosis, treatments, disabilities, allergies, blood pressure values, family history, medication list, lab results, and progress over time, as well as record notes from hospital and primary care visits.

*“Possibly information about which tests/samples they [the care professionals at hospital] have taken and such things. What could happen to you and how you [your health condition] will be in the future …?”*

Participants wished to have access to their medication list, contraindications, and possible side effects. In addition, participants wanted more administrative information; current prescriptions, the last date for retrieving their medication from the pharmacy, as well as pharmacy rules and regulations regarding medications.

##### An overview of risk factors and disabilities

Participants wished to have information about risk factors and preventive activities, e.g. how their smoking, alcohol consumption, exercise, and food habits affect their health and risk of recurring strokes. However, the timing of this information is essential, and it is important to focus on risk factors at a later stage in the recovery process.

*“As a patient you need this information. But the question is how receptive you are, e.g. I know that smoking is a risk factor, but it is also about weight loss and so on. At first, I think you are more focused on returning to life again. But later you become interested in preventing another stroke, to keep from ending up in the same situation again.”*

Participants experienced it as challenging to understand their invisible disabilities such as chronic fatigue, cognitive impairments, and personality changes and to explain them to others. Patients requested a simple description of their disabilities that they can share with e.g. their next-of-kin.

*“Just to know that these disabilities exist is good. But there are many disabilities and [it is difficult] to describe them in a way so that relatives understand you.”*

*“All these disabilities do not necessarily appear at the same time, so there is no certainty that they will appear just as you get discharged from the hospital. They might come a little later. But people can be prepared that they might appear in some way. So a compilation [of the disabilities] could be helpful.”*

##### Measurement and documentation of health related parameters

Some of the participants were interested in tracking personal observations by measuring both physical parameters e.g. blood pressure and sugar level, and by documenting subjective assessments of e.g. fatigue and mood history to understand their illness.

### Collaboration with care providers

Participants were interested in improved communication with different care providers. They experienced a lack of information about e.g. different caregivers contact information and expertise.

#### An overview of care providers’ contact information, their specialities and responsibilities

For many participants, the roles and responsibilities of different healthcare professionals were unclear as they had never been in contact with e.g. a speech language therapist or a counsellor before. Participants were therefore interested in having information about the different care professionals in a neuro team and professionals in primary care centres, their roles and responsibilities so as to know who to contact about what issues, and of course support in how to contact them. Participants also talked about their struggle for contacting a caregiver for receiving rehabilitation once the initial rehabilitation provided by the neurology team was over. Another problem experienced by the participants was that not all healthcare professionals in primary care were experienced in working with stroke patients, and participants wanted to be able to make an informed choice in selecting the caregivers that better suit their needs.

*“The reason that patients are a little dissatisfied with their primary care physicians is that they think that the physicians may not know a lot about stroke or diabetes. This kind of information, as a patient, you would like to have, or information about which primary care centre has a little more knowledge about my problem.”*

#### Support for sharing of personal observations

Communicating their health issues and questions to healthcare professionals was also experienced as a problem. As described above, some participants were interested in documenting personal observations, but they also wanted to be able to share this data with their physician as input to their meetings. In addition, participants wished to have support for preparing health related questions since remembering to ask the right questions during brief meetings with healthcare was a challenge for them.

“*But one of those diaries is great to have, before you visit a doctor, you can add up questions, and write a little bit about your problems.”*

“*When you want go to the doctor in one week or in 14 days, you might think that “I will remember to ask about it, but it is forgotten the day you get there [at the doctor’s office]”*

#### Tracking the rehabilitation process

In this study rehabilitation refers to a cyclical process of identifying the patients’ needs, defining the realistic goals, activities and actions for achieving the goals and assessment of progress against the agreed goals [33]. Although rehabilitation has a great importance in the motor and cognitive recovery process after stroke, the participants in this study experienced a lack of information about tracking their rehabilitation processes, their disabilities and related assistive devices.

*“Me, who suffer from stroke, have to be the unifying link in my rehabilitation - it is hard”*

##### An overview of goals and planned activities

The participants generally experienced a lack of rehabilitation throughout the care process. In addition, none of the patients had experience of being provided with a rehabilitation plan either at rehabilitation clinics or at home. Patients wished to have access to information about their rehabilitation such as goals, planned activities, and follow up efforts. Patients experienced the need of having access to a rehabilitation plan where they can identify their problems together with a neuro team, and set their personal rehabilitation goals and plan and track activities. Patients also indicated that they would like to have access to e.g. online exercises and instructions to necessary activities related to their goals.

#### Practical guidance through healthcare and community services

Indications were that participants searched for a holistic, practical guidance through healthcare. The participants were also interested in peer support from other patients in similar situation for finding coping strategies throughout the care and rehabilitation processes.

##### Rights and responsibilities regarding e.g. continued rehabilitation, assistive devices and health insurance

The participants showed an interest in understanding the rights and responsibilities they have regarding e.g. assistive devices and rehabilitation. However, they expressed that it would be desirable if they together with their next-of-kin could review this information later on at home as they receive lots of information at the hospital that they are not able to process. Participants mentioned that information about housing adjustments might not be as important as information about rehabilitation efforts for patients who receive rehabilitation at different rehabilitation clinics after discharge from hospital. They meant this information will be relevant when they return home either from hospital or from a rehabilitation clinic.

*“This part is important, where to go and [whom to contact] and what my rights are…”*

Participants expressed that having access to an overview of available assistive devices related to their disabilities after a stroke and the practicalities related to gaining access, e.g. cost and availability, is of great importance to them.

*“I really do not know which assistive devices I need at home. Just to see what is out there. I mean e.g. how I get into the bathtub; there is a bathtub that has side doors that you can step into. I mean that stuff is good to know.”*

Participants experienced a lack of information about applying for a specific device and the organisation the application should be sent. They wished to be able to track the process and receive information about rejection or approval of the application.

In addition, the participants expressed that having access to information about different private companies and manufacturers and retailers of assistive devices would also be of great importance. Participants also wished to have access mainly to the general information e.g. contact information and general regulations regarding their social health insurance as well as information regarding different private health insurance alternatives. The participants experienced a need of having access to information about counsellors and legal representatives who can help them in issues and questions regarding insurances.

##### An overview of patient associations and social networks

Currently, the contact information of patient associations in Stockholm is provided at the hospital where the patient receives care and rehabilitation. However, participants were interested in having more complete description of different patient associations and social networks.

### Recommendations and implications for design

Based on the modelled patient journey and identified issues patients’ experience throughout the journey, their information needs and preferences, researchers in this study have suggested potential eHealth services and recommendations about stroke specific design implications. The eHealth services and details on required features/functionalities to meet patients’ needs are illustrated in Table 3.

**Table 3:** An overview of potential eHealth services and functionalities

Patients’ information needs	Potential eHealth services	Required functionalities
An overview of past events	My discharge notes	Recordings of the discharge meeting
		Translation of medical terms/statements to plain language
An overview of planned events	My calendar	Planned/booked appointments with care professionals
		Care professionals who should contact me
		Sharing calendar with the next-of-kin
		Reminders about appointments
	My referrals	Current status of referrals sent to different care providers
An overview of health related information	My health information	Access to information from electronic health record system in an understandable way
		Diagnosis
		Lab results
		Visualisation of my progress over time
		Information about my current and resolved health problems, as well as common problems that may occur
		Reminders about preventive actions
	My medication	An overview of my current medications and possible drug interactions
		A description of my medication and possible side effects
		Reminders about renewal of my prescription
		Reminders about the last date I can get my medication from pharmacy
		Links to existing resources such as national online medication and treatment guidelines resources (in Sweden 1177 and Pharmaceutical specialities in Sweden)
An overview of risk factors and disabilities	My risk factors	A list of my risk factors
		Preventive actions
		Reminders about my preventive actions
	My disabilities	A list of my earlier, resolved disabilities
		An understandable description of my current disabilities
An overview of care providers’ contact information, their specialities and responsibilities	My caregivers	My current caregivers
		Parameters for choosing care provider (e.g. specialisation, cost, geographic location, patient reviews)
		Links to different care providers centres websites
		Patient assessments/reviews of primary care centres regarding stroke care
		An overview of neurology teams and other care professionals who are experts in stroke
Measurement and documentation of health related parameters	My diary	My personal observations
		My health condition in relation to my medication
		Self-monitoring of e.g. weight, blood pressure
Support for sharing of personal observations		Surveys/questionnaires to facilitate documentation of health condition and mood history
		Sharing information with care professionals e.g. before a revisit
An overview of the goals and planned activities	My rehabilitation	Tools for defining goals, activities and follow up efforts
Rights and responsibilities regarding e.g. continued rehabilitation, assistive devices and health insurance	My rights & my responsibilities	Access to information about the healthcare guarantee (care within a certain period of time)
		Support for choice of caregivers that suit the patient’s need better
	My assistive devices	A list of available tools
		Devices that I have been granted
		Private alternatives for purchase of assistive devices
	My insurances	General information about insurance companies
		Reminder about obtaining insurance
An overview of patients associations and social networks	My patient organisations and support associations	A list of available patient organisations and support associations
		Link to stroke patient associations
		Access to social networks

**Fig. 4:**
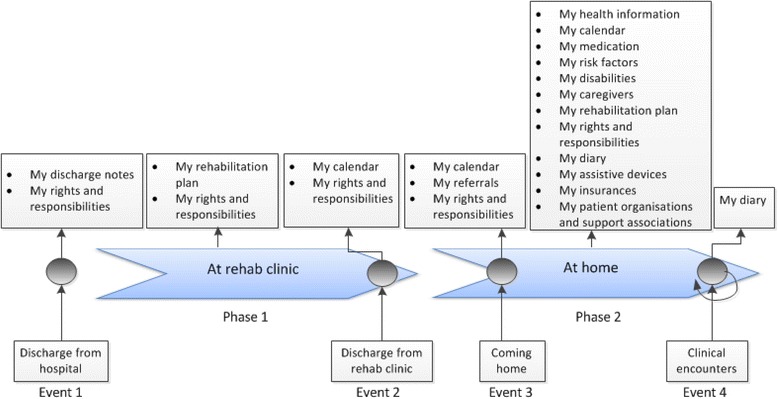
An overview of potential eHealth services in relation to the patient journey

The suggested eHealth services in relation to the patients’ information needs throughout the patient journey were discussed in the focus groups and the results are presented in Fig. 4.

The results highlight the considerable amount of information (practical and clinical) that patients need at home. At hospital or rehabilitation centres most patients are not physically or mentally prepared to receive information. Once they return home and want to follow their care and rehabilitation processes, they experience a lack of necessary information. To support the patients in managing their illness it is therefore imperative to give them an overview of what happened during the hospital stay and what is planned after discharge from hospital. While some needs change over time throughout the care and rehabilitation processes other needs remain stable. Patients wish to know about their rights and responsibilities regarding assistive tools, rehabilitation efforts, and choice of caregivers directly after discharge from hospital. A rehabilitation plan in which they can access their rehabilitation needs, goals, and activities related to their goals and the progress over time is also needed both at the rehab centres and at home.

#### Stroke specific design implications

Some recommendations about functionalities that are common for many eHealth services are presented in more detail.

##### Reminders

Reminders for e.g. appointments with care professionals, renewal of prescriptions, for medications, and also for preventive actions are essential in design of eHealth services that aim to allow stroke patients to track and manage their disease.

##### Information sharing

Patients should be able to share information about their clinical appointments with their next-of-kin. In addition, patients should have possibility to share their mood history, problems related to their medications, and health problems with care professionals e.g. before a revisit. Thus, the care professional has the opportunity to review the information sent by the patient before the visit and be able to devote more time for the patient and the encounter.

##### Integration between eHealth services and links existing health related sources

Connection between different eHealth services will give the patient possibility to move between different services and identify e.g. the relation between a health condition and medications. In addition, links between eHealth services and other existing national online medication and treatment guidelines sources such as Pharmaceutical Specialities in Sweden and 1177 which contain a summary of medication facts, treatments and also information about preventive care and care organisation in Sweden will help patients to access medication related information if necessary. Furthermore, links to sources about assistive devices, insurance companies, caregivers’ websites, etc. will give patients access to additional information if necessary. A lot of information is already available online, but it is a challenge for stroke patients to find it – curating it in through links in one place would be useful. In addition, the eHealth services should be integrated with the patients’ electronic health record system for access to medical and health related information such as diagnosis, lab results and referrals.

##### Repetition of information

Repetition of information is of great importance as patients and their next-of-kin are in a state of shock immediately after the stroke and has difficulty to understand all information they receive from care professionals at the hospital. Therefore, video recording the discharge meetings with subtitles from the hospital or the rehabilitation centre where patients has received care and rehabilitation will give patients and their next-of-kin access to the information even a long time after discharge. Some information is produced and provided at the hospital but it is also needed for subsequent care and rehabilitation at home.

Having suffered a stroke, affects your ability to process information and it is not uncommon for stroke patients to be unconscious or suffer memory loss from the acute phase of the illness trajectory. A photo diary from the time at the hospital would therefore be useful for both patients and their next-of-kin at home.

##### Understanding the information

Information about e.g. diagnosis, treatment, medications should be documented and provided in an understandable manner to patients and their next-of-kin.

A comprehensive overview of patients’ risk factors and disabilities will provide a common understanding of patient’s health condition and behaviour for both patients and their next-of-kin. A list of appropriate devices that are related to patients’ disabilities will support patients and their next-of-kin to choose an assistive device that suit the patient’s needs better.

## Discussion

We used the patient journey model to understand the processes stroke patients go through after discharge from the hospital and the problems they experience during this journey. Gaining this insight is important input to proposing new eHealth services for post-discharge stroke patients.

The patient journey model identified in this study matches the stroke illness trajectory framework described by Kirkevold [20]. The stroke illness trajectory is based on the Chronic Illness Trajectory Framework developed by Corbin and Strauss, but rather than the 8 phases in the chronic illness trajectory, it consists of four phases: ‘Trajectory onset’, ‘Initial rehab’, ‘Continued rehab’ and ‘Semi-Stable’. In this study, we have only focused on the latter phases as they are relevant to post-discharge stroke patients. The two phases identified in this study, namely ‘At rehab clinic’ and ‘At home’ cover the three identified phases in the trajectory framework since for the patient there is a very unclear boundary between the continued rehab and semi-stable phases. However, we have identified four main events directly after discharge from hospital. The main events within the phase ‘At home’ identified in the patient journey model also cover the events such as home visits, rehabilitation planning, and physician appointments in our previous study [23].

Patients’ experiences and information needs explored in this study show a lack of practical/administrative information such as patient’s rights regarding assistive devices, rehabilitation efforts, healthcare guarantee (care within a certain period of time), housing adjustments, choice of caregivers, and general information about insurance companies. Patients also experienced a need of clinical information about e.g. rehabilitation efforts, medication lists, referrals, diagnosis, and lab results. These results confirm findings of previous studies where stroke patients unmet information needs had been investigated [34, 35]. This is however not unknown within the field of caring and nursing science; already in 1998 Wiles et al divided appropriate information to stroke patients into ‘clinical information’, ‘practical information’, and ‘information on continuing care and resources in the community’[36]. Despite this, stroke patients still suffer a lack of appropriate solutions to provide them with this information. Today we have the opportunity to use ICT and mobile technology to design appropriate eHealth services that can meet patients’ needs throughout the care and rehabilitation processes. Currently, eHealth services for patients are developed and implemented worldwide, often in the form of personal health records (PHRs). In Sweden, patients’ online access to their full electronic health records is currently rolled out nationally [37], and in the OpenNotes project patients have been given online access to their primary care physicians’ notes [38]. Such solutions meet many of the needs with respect to clinical information identified in this study and others. Yet, these solutions are often limited to the information that is currently available within healthcare’s information systems, and despite being a very good starting point, it will not meet the full needs of the stroke patients as identified in this study. However, eHealth services proposed in this study need to be integrated to the current eHealth infrastructure to ensure that patients have access to one entrance point to their health information. In addition, our results in comparison with the trajectory illness framework and stroke illness trajectory indicate a change of patients’ needs throughout the post-discharge stroke care and rehabilitation as patients move from one phase to another one. There are trigger events such as home visits, rehabilitation planning, doctor appointments etc. within the phases where patients need supportive eHealth services [23]. Therefore, it is imperative to design and implement future eHealth services that not only meet the full range of patients information needs, but also support their different needs at different stages throughout their journey.

The proposed eHealth services in Table 3 are not an exclusive list, but rather suggestions based on the participants’ experiences in the focus groups. Anyone reading about the problems may come up with their own ideas for eHealth services that could improve the situations for stroke survivors. This is one of the strengths of using patient journey mapping and patient experiences as a basis for design.

A potential weakness of this study is that participants might not be representative of many stroke patients as they had milder disabilities and were able to use computers. Another limitation of this study is the low number of participants involved in the focus groups. Still, even this limited case study points to important insights into the patients’ experiences, and important ideas for eHealth services were produced. All participants were recruited locally, and had received their care within the same county council. This may limit the transferability of the results in terms of the patients’ experiences and some of the proposed eHealth services. Yet, as a designer of eHealth services one may recognize similar problems from other contexts where these results can be applied. An important, transferable, result of this study is however the method of using patients’ experiences as a basis for proposing new eHealth services to improve healthcare from a patient perspective. The approach can be applied locally in any context to explore patients’ experiences and propose solutions to identified problems.

Another potential weakness is that participants in both groups (those who had stroke more than 10 years and also participants who suffered stroke less than 10 years) did not have any experience of rehabilitation planning. This may be due to that some participants who were over 65 years old in two focus groups did not remember having any experience with rehabilitation planning. This may also be due to the lack of appropriate eHealth services at the time the participants had their stroke, or the fact that they all suffered their stroke many years ago and the organisation with neurology teams is fairly new. Regardless of the explanation, the analysis of the patients’ needs indicate that a rehabilitation plan is needed at different phases throughout the patient journey. In addition, the fact that information technology has been developed considerably during last decades makes the design of supportive eHealth services possible.

Finally, usability and interface design must be mentioned when discussing design of eHealth services for stroke patients. The design needs to be adaptable to different stroke patients’ disabilities, which is a challenge in itself, but even if the design is as simple as possible, there will still be patients who are not able to use the services due to a lack of digital literacy. Next-of-kin may however play an important part as a proxy for the patient in these cases. Several studies highlighted the impact of stroke on patients’ next-of-kin and the importance of their role in the patients’ journey [39–41]. In addition studies stressed the unmet information needs of next-of-kin and also a lack of support for family members [42–46]. Several of the participants in our focus groups also mentioned family members as essential both during the acute phase and later during post-discharge rehabilitation. Therefore it is imperative to identify the information needs of next-of-kin of stroke patients and to design necessary eHealth services for them.

In addition, there may be patients that are simply not interested in using the eHealth services and prefer having paper-based information and physical contacts with the care professionals. Therefore, it is important to ensure that the eHealth services are designed as complementary to existing services and resources, and to facilitate access to care and rehabilitation for as many patients as possible.

## Conclusion

Patient journey models and qualitative analysis of patients’ experiences are powerful tools that can be used to improve healthcare from a patient perspective. In this study we show how such tools can be used as input to the design of eHealth services, but by creating a patient journey model and describing patients’ experiences of going through this journey, we also create opportunities for reaching a common understanding of issues and problems experienced by patients, thereby facilitating improvement work and in the long run increased patient satisfaction. Patients’ need of different eHealth services changes as their experience of the illness and their information needs change. Although it is challenging to design appropriate eHealth services that intend to meet the patient’s needs throughout the patient’s journey, an in-depth analysis of patients’ needs will facilitate the design of services that are useful at several phases in the patient’s care and rehabilitation processes.

The main categories identified in this study indicate that patients not only need to have access to health related information about their care and rehabilitation processes but also practical guidance through healthcare and community services. The eHealth services should therefore focus on giving access to necessary information to meet the patient’s different needs. In addition, since stroke patients suffer from different (physical, psychosocial, and cognitive) disabilities due to stroke, designing appropriate eHealth services requires an understanding of these disabilities.

## Abbreviations

ICT, information and communication technology; PHR, personal health record
